# Relevance of serum interleukin-33 and ST2 levels and the natural course of chronic hepatitis B virus infection

**DOI:** 10.1186/s12879-016-1543-x

**Published:** 2016-05-16

**Authors:** Shu-Ling Huan, Ji-Guang Zhao, Zhen-Li Wang, Shuai Gao, Kai Wang

**Affiliations:** Department of Hepatology, Qilu Hospital of Shandong University, Jinan, 250012 Shandong China; Department of clinical laboratory, Qingdao Municipal Infectious Disease Hospital, Qingdao, 266033 Shandong China; Institute of Hepatology, Shandong University, Wenhuaxi Road 107#, Jinan, 250012 Shandong China

**Keywords:** Chronic hepatitis B, Interleukin-33(IL-33), ST2, Natural course, Enzyme-linked immunosorbent assay (ELISA)

## Abstract

**Background:**

Interleukin-33 (IL-33) and ST2 have been demonstrated to be associated with liver damage. However, their potential value in hepatitis B virus (HBV) infection remains unknown. This study was designed to investigate the change of serum IL-33 and ST2 levels in the natural course of chronic HBV infection.

**Methods:**

A total of 120 patients with chronic hepatitis B (CHB), 20 chronic hepatitis B virus carriers in immunotolerant phase and 28 healthy controls were enrolled in this study. All patients with CHB were divided into four groups according to their serum ALT levels. The serum levels of IL-33 and ST2 of all participants were determined by enzyme-linked immunosorbent assay, and compared between each two out of those six groups.

**Results:**

No significant differences were found in serum levels of IL-33 and ST2 between the group of CHB with ALT 1–2 upper limit of normal and the healthy controls (*P* = 0.354 for IL-33 and *P* = 0.815 for ST2). Other than that, there were significant differences when serum levels of IL-33 and ST2 were compared between any other two out of those six groups (*P* < 0.05, respectively). The overall correlation analysis indicated that changes of serum IL-33 and ST2 levels were positively associated with ALT levels in patients with chronic HBV infection (*rs* = 0.879, *P* < 0.001 for IL-33 and *rs* = 0.923, *P* < 0.001 for ST2). No significant differences were found when the serum levels of ALT, IL-33 and ST2 were compared between patients with HBeAg-positive CHB and HBeAg-negative CHB.

**Conclusions:**

Our study revealed that the serum levels of IL-33 and ST2 varied in different courses of chronic hepatitis B virus infection. The serum levels of IL-33 and ST2 elevated as serum ALT levels increased in patients with CHB. They might indicate liver damage for patients with CHB, just like ALT.

## Background

Interleukin-33 (IL-33), a new member of the IL-1 family, induced the production of pro-inflammatory and T helper-2 (Th2)-associated cytokines. ST2 was to weaken Th2 inflammatory responses as its receptor [1]. Hepatitis B virus (HBV) infection is still a major health problem worldwide. There are approximately 350 million people chronically infected with HBV globally, and they are at great risk of developing liver cirrhosis and hepatocellular carcinoma. About 5 % of adulthood and over 90 % of infants and young children who are infected with HBV will evolve chronicity [2]. Many patients with chronic hepatitis B (CHB) ultimately progress to liver cirrhosis and hepatocellular carcinoma [3, 4]. Previous studies showed that the interaction of HBV, hepatocytes and the host immune system determines the persistence of HBV infection and chronic inflammation [5]. The immune system was known to be suppressed by the viral infection and related hepatocyte injuries [6, 7]. Although experimental evidence suggested that antigen-specific Th1 immunity and pro-inflammatory cytokines played an important role in the HBV related liver injury and clearance of viruses [8, 9], serum IL-33 levels were associated with liver damage of patients with CHB [10], serum levels of IL-33 and soluble ST2 elevated in liver failure, which could be a sign of immune hyperactivation, and/or a mechanism to down-regulate inflammation [1]. Soluble ST2 may be useful to discern acute from chronic hepatic failure or to monitor the course and the severity of the disease. It is widely accepted that the adaptive immune responses play major roles in the clearance of HBV infection. However, the role of innate immunity during HBV infection appears not to be well understood, which can be attributed to the fact that the recruitment of patients in the very early, asymptomatic phase of HBV infection is very difficult [11, 12].

Remarkable progress had been made in the understanding of the natural history of chronic HBV infection. The natural course of chronic HBV infection was perceived as consisting of 4 phases: immune tolerance, immune clearance (HBeAg-positive chronic hepatitis B), inactive carrier state, and reactivation (HBeAg-negative chronic hepatitis B) [13]. Although serum levels of IL-33 and ST2 were up-regulated in liver failure and CHB, the relevance of serum levels of both molecules and patients with hepatitis B virus infection needed further research and serum alanine aminotransferase (ALT) activity is an important marker for liver damage in patients with CHB. We therefore sought to investigate the serum measurements of IL-33 and ST2 in patients with chronic hepatitis B virus infection and study the relationship of IL-33 and ST2 with ALT and HBeAg. We examined the levels of serum IL-33, ST2 and ALT in patients with CHB, chronic hepatitis B virus carriers in immunotolerant phase and healthy controls (HC) so that we could determine whether IL-33 and ST2 had relevance with chronic HBV infection in our study.

## Methods

### Patients

A total of 120 patients with CHB, 20 chronic hepatitis B virus carriers in immunotolerant phase, and 28 HC were successively conscribed at the outpatient and inpatient services in Qilu Hospital of Shandong University and Qingdao Municipal Infectious Disease Hospital from September 2012 through May 2014. The patients with CHB and chronic hepatitis B virus carriers in immunotolerant phase were defined according to the criteria of Asian-Pacific consensus statement on the management of chronic hepatitis B [14]. All patients with CHB were stratified into four groups according to their serum levels of ALT, including ALT1-2 upper limit of normal (ULN), ALT2-5ULN, ALT5-10ULN, ALT > 10ULN. The number of patients with CHB in each group was calculated based on the data obtained from preliminary experiment by using PASS 11. Besides, all patients with CHB were divided into two groups of 65 patients with HBeAg-positive chronic hepatitis B and 55 patients with HBeAg-negative chronic hepatitis B according to their serum HBeAg. Patients with chronic HBV infection were confirmed positive for HBsAg, HBeAg or HBeAb and detectable HBV virus for at least 12 months [13]. People who had comorbidities such as history of infection, hodiernal hepatitis A, C, D and E viruse infection, human immunodeficiency virus infection, autoimmune hepatitis, metabolic liver disease et al., were eliminated. All patients had not received any therapy of liver injury or been exposed to obvious liver damage compound.

Written informed consent was obtained from individual participants, and the study was approved by Ethical Committee of Qilu Hospital of Shandong University and Qingdao Municipal Infectious Disease Hospital.

### Laboratory assays

Sera were extracted after blood samples were taken from all the participants and then stored at −80 °C till needed. The measurements of serum IL-33 and ST2 in all participants were determined by enzyme-linked immunosorbent assay (ELISA) using human IL-33 and ST2 ELISA Kits (Immunoway Biotechnology, USA) according to the manufacturer’s instruction. The measurements of serum IL-33/ST2 in individual samples were calculated according to the standard curve established using the recombinant IL-33 and ST2 provided.

HBV-related HBsAg, HBsAb, HBcAb, HBeAg, and HBeAb were detected by an electro-chemiluminescence immunoassay using Roche kits (Roche Diagnostics, Germany) according to the manufacturer’s instruction. The level of serum ALT was detected by Velocity method using big biochemistry automatic analyzer (Olympus 2700, Japan).

The serum HBVDNA levels were measured by nested reverse transcription polymerase chain reaction (PCR) quantitative assay using a luciferase quantitization detection kit, following the protocols (Roche Amplicor, Germany). The detection limit of viral DNA was 300 copies/ml [12, 15].

### Statistical analysis

The data were expressed as the median (inter-quartile range). The differences between the groups were analyzed by Mann-Whitney *U*-test and overall correlation between ALT and IL-33, as well as ST2 were analyzed by Spearman’s rank correlation test using SPSS17.0 software. A two-side *P* value of < 0.05 was considered statistically significant.

## Results

There were no significant differences in the distribution of age or gender among the six groups of participants, and there were no significant differences in the serum HBVDNA levels among the five groups of chronic HBV infection (Table 1).

**Table 1:** The demographic and clinical features of all the participants

Parameters	CHB(ALT1-2ULN)	CHB(ALT2-5ULN)	CHB(ALT5-10ULN)	CHB(ALT ≥ 10ULN)	CHBVIT	HC
NO.	30	30	30	30	20	28
Age (years)						
Median (inter-quartile range)	36.0 (26.0–41.5)	32.5 (25.8–37.5)	33.0 (25.8–38.5)	36.5 (26.0–42.0)	32.0 (27.3–35.8)	32.0 (24.0–37.5)
Sex N (%)						
Male	18 (60)	20 (67)	21 (70)	19 (64)	12 (67)	18 (64)
Female	12 (40)	10 (33)	9 (30)	11 (36)	8 (33)	10 (36)
HBVDNA (log10 copies/mL)						
Median (inter-quartile range)	7.4 (6.2–7.6)	7.3 (6.1–7.5)	6.4 (5.7–7.0)	6.3 (5.7–6.6)	7.5 (6.7–7.7)	NA
HBeAg, pos/neg	15/15	16/14	18/12	16/14	20/0	0/28

Normal values: ALT: 1ULN = 40 IU/L. The lower limit of detection: HBVDNA 300 copies/ml. Data were expressed as median and inter-quartile range or arithmetic mean and standard deviation (SD)

*ALT* alanine aminotransferase, *CHB* chronic hepatitis B, *CHBVIT* chronic hepatitis B virus carriers in immuno-tolerant phase, *HBeAg* hepatitis B e antigen, *HBsAg* hepatitis B surface antigen, *HBV DNA* hepatitis B virus DNA; *HC* healthy control, *ULN*, upper limit of normal

**Fig. 1:**
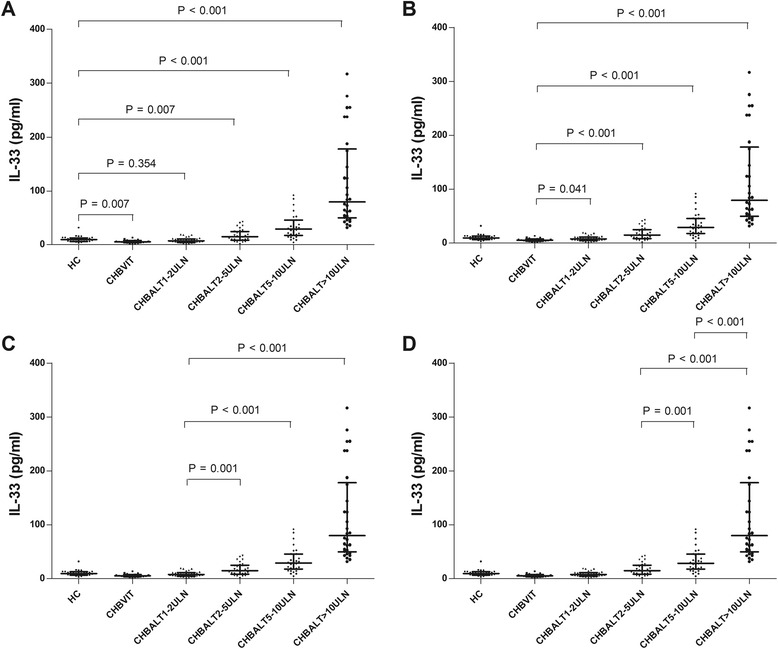
**a** Comparsions of IL-33 between healthy controls (HC) and the rest five groups. **b** Comparisons of IL-33 between chronic hepatitis B virus carriers in immuno-tolerant phase (CHBVIT) and the four groups of patients with CHB. **c** Comparisons of IL-33 between CHBALT1-2ULN group and the rest three groups of patients with CHB. **d** Comparisons of IL-33 between CHBALT2-5ULN group and CHBALT5-10ULN group, between CHBALT2-5ULN group and CHBALT > 10ULN group, between CHBALT5-10ULN group and CHBALT > 10ULN group

**Fig. 2:**
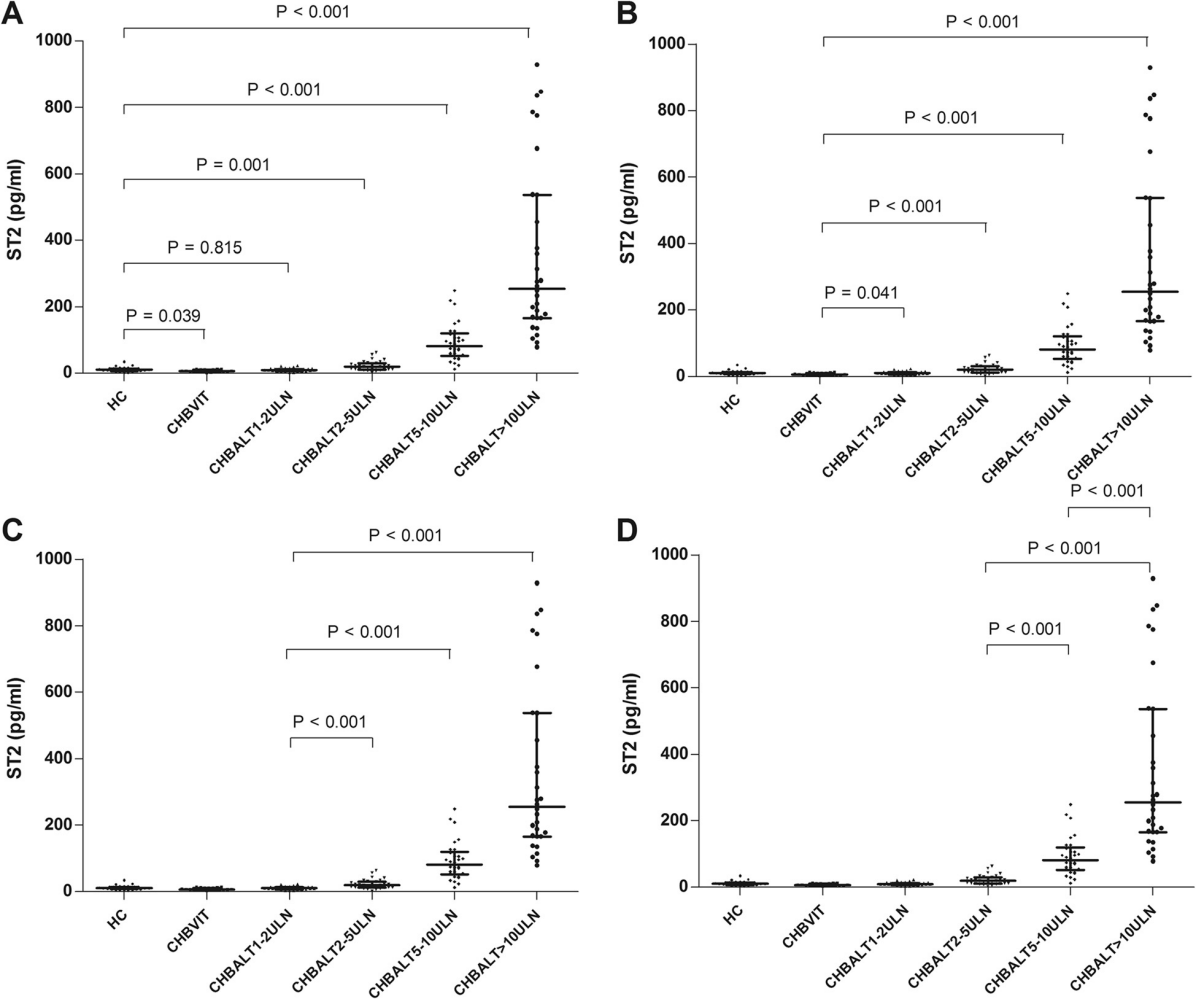
**a** Comparisons of ST2 between healthy controls (HC) and the rest five groups. **b** Comparisons of ST2 between chronic hepatitis B virus carriers in immuno-tolerant phase (CHBVIT) and the four groups of patients with CHB. **c** Comparisons of ST2 between CHBALT1-2ULN and the rest three groups of patients with CHB. **d** Comparisons of ST2 between CHBALT2-5ULN group and CHBALT5-10ULN group, between CHBALT2-5ULN group and CHBALT > 10ULN group, between CHBALT5-10ULN group and CHBALT > 10ULN group

The serum levels of ALT, IL-33 and ST2 in the six groups of participants were listed in Table 2. The serum levels of IL-33 and ST2 were compared between each two out of those six groups. No significant difference was found in serum IL-33 levels between the CHB ALT1-2ULN group and the HC (*P* = 0.354). Other than that, there were significant differences when serum IL-33 levels were compared between any other two out of those six groups (*P* < 0.05, respectively). Although the *P* values were significant in above comparisons, the direction of the difference varied. IL-33 levels were significantly higher in healthy control group compared to the group in immunotolerant phase but it was significantly lower in healthy control group compared to the groups with ALT >2 times high (Fig. 1). In terms of serum ST2 concentrations, a similar trend was also observed. There were significant differences in serum ST2 levels between any other two out of those six groups (*P* < 0.05, respectively), except for those between the CHB ALT1-2ULN group and the HC (*P* = 0.815). Although the *P* values were significant in above comparisons, the direction of the difference varied. ST2 levels were significantly higher in healthy control group compared to the group in immunotolerant phase but it was significantly lower in healthy control group compared to the groups with ALT >2 times high (Fig. 2).

**Table 2:** Serum levels of ALT, IL-33 and ST2

Groups	NO.	ALT (IU/L)	IL-33 (pg/ml)	ST2 (pg/ml)
HC	28	26.0 (23.3–31.5)	9.6 (6.6–12.7)	10.6 (6.5–13.6)
CHBVIT	20	26.5 (22.5–34.8)	5.3 (3.6–7.9)	6.1 (3.7–10.8)
CHB (ALT1-2ULN)	30	57.5 (49.0–69.8)	7.8 (4.5–10.9)	9.9 (6.2–13.2)
CHB (ALT2-5ULN)	30	117.0 (95.0–145.3)	14.8 (8.4–24.6)	19.7 (11.8–30.2)
CHB (ALT5-10ULN)	30	268.0 (237.5–319.5)	29.9 (11.8–21.8)	80.9 (33.6–69.7)
CHB (ALT ≥ 10ULN)	30	1005.0 (637.0–1558.5)	79.5 (49.7–177.9)	255.4 (165.8–536.6)

Normal values: ALT: 1ULN = 40 IU/L. Data were expressed as median (inter-quartile range)

*ALT* alanine aminotransferase, *CHB* chronic hepatitis B, *CHBVIT* chronic hepatitis B virus carriers in immuno-tolerant phase, *HC* healthy control, *ULN* upper limit of normal

**Fig. 3:**
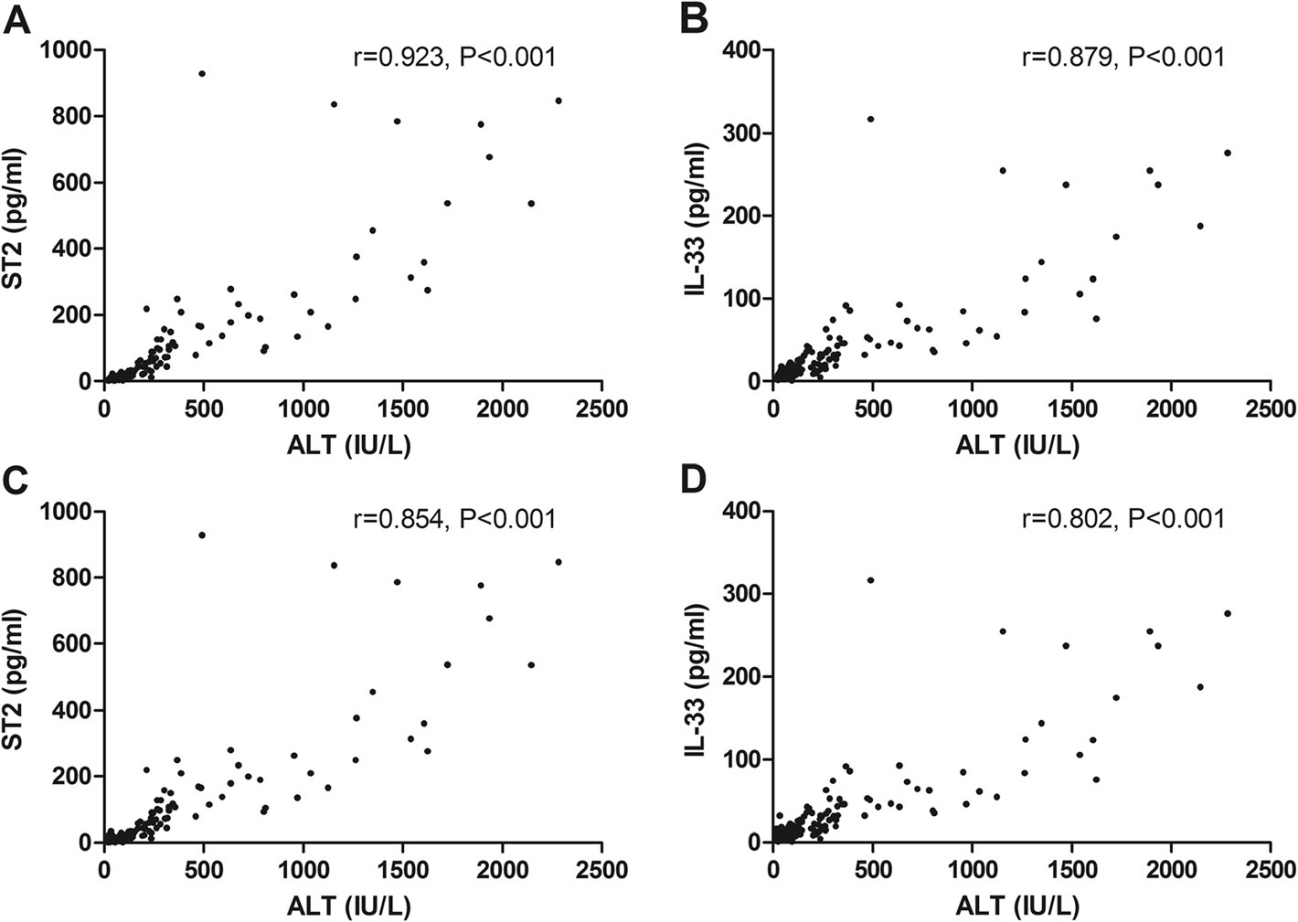
**a** The overall correlation between ALT and IL-33 in patients with chronic HBV infection. **b** The overall correlation between ALT and ST2 in patients with chronic HBV infection. **c** The overall correlation between ALT and IL-33 in healthy controls. **d** The overall correlation between ALT and ST2 in healthy controls

Next, the overall correlations between serum ALT and IL-33, as well as ST2 were analyzed. The result indicated that changes of serum IL-33 and ST2 levels were positively associated with ALT levels in patients with chronic HBV infection (*rs* = 0.879, *P* < 0.001 for IL-33; *rs* = 0.923, *P* < 0.001 for ST2). However, there were no significant correlations between ALT and IL-33, or ST2 in healthy controls (*rs* = −0.134, *P* = 0.497 for IL-33; *rs* = −0.012, *P* = 0.952 for ST2) (Fig. 3).

**Fig. 4:**
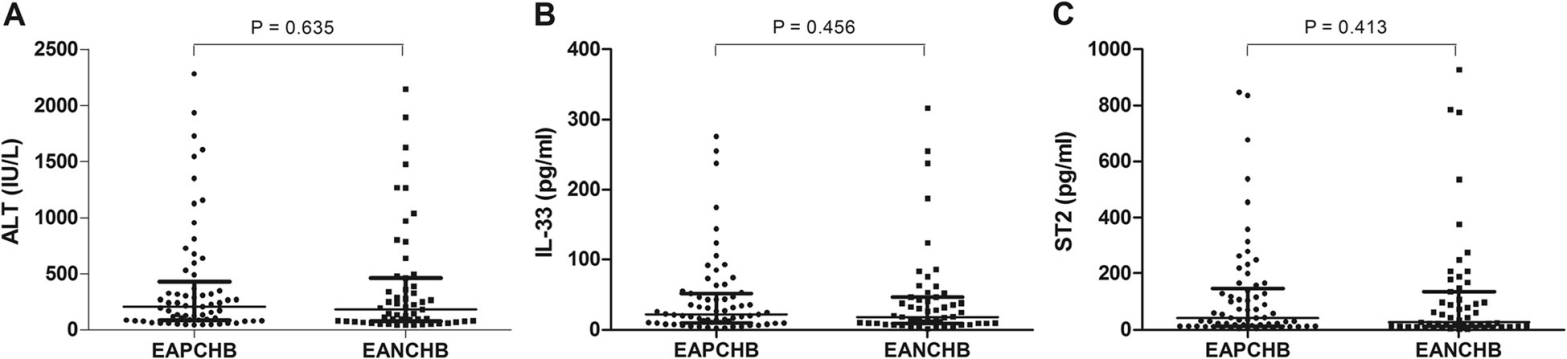
**a** Comparisons of ALT between EAPCHB (HBeAg-positive chronic hepatitis B) group and EANCHB (HBeAg-negative chronic hepatitis B) group. **b** Comparisons of IL-33 between EAPCHB group and EANCHB group. **c** Comparisons of ST2 between EAPCHB group and EANCHB group

Finally, when the levels of serum ALT [207 (84.5, 427.5) vs 182 (76, 460)], IL-33 [22.13 (9.69, 51.57) vs 18.13 (8.76, 45.7)] and ST2 [41.78 (12.49, 147.20) vs 26.58 (11.32, 134.52)] were compared between the group with HBeAg-positive chronic hepatitis B and the group with HBeAg-negative chronic hepatitis B, no significant differences were found (*P* = 0.635 for ALT, *P* = 0.456 for IL-33, and *P* = 0.413 for ST2) (Fig. 4).

## Discussions

The natural course of chronic HBV infection had been treated as being comprised of 4 phases: immune tolerance, immune clearance (HBeAg-positive chronic hepatitis B), inactive carrier state, and reactivation (HBeAg-negative chronic hepatitis B). Understanding the dynamic nature of chronic HBV infection was crucial in the management of HBV carriers and underscored the need for long-term monitoring [13]. IL-33 was a member of the IL-1 family by classics appraisal. ST2 is a receptor for IL-33. Their multiple variants like ST2L or ST2V exist through alternative splicing. The main biological properties of IL-33 were to drive production of pro-inflammatory and Th2-associated cytokines in mast cells and Th2 lymphocytes [16, 17].

Aditionally, recent evidence suggested that IL-33 belonged to the larger family of damage-associated molecular pattern molecules and functioned as an “alarmin” similar to high-mobility-group-protein B1 (HMGB1) [16]. ST2 stimulated MYD88, IRAK1, IRAK4, and TRAF6 by phosphorylation of MAPK3/ERK1 and/or MAPK1/ERK2, MAPK14, and MAPK8, Whereas ST2L exerted pro-inflammatory effects of IL-33. Increased serum levels of soluble ST2 had been reported in conditions like sepsis and dengue virus infection [18, 19]. Moreover, in murine inflammatory models, the production of pro-inflammatory cytokines preceded ST2 expression [20, 21]. Additionally, soluble ST2 could diminish the formation of inflammatory mediators [21, 22]. Hepatic over-expression of IL-33 had been detected in patients with liver fibrosis, chronic hepatitis B and hepatic failure. A previous study provided evidence for elevated levels of IL-33 and soluble ST2 in liver failure, which could be a sign of immune hyperactivation, and/or a mechanism to down-regulate inflammation. Especially, soluble ST2 may be useful to discern acute from chronic hepatic failure or to monitor the course and the severity of the disease [1]. Recent studies revealed that the levels of serum IL-33 were elevated in patients with chronic hepatitis C and were dropped significantly after treatment with interferon, and Wang et al. found that there was a significant correlation between IL-33 and ALT concentration in chronic hepatitis C [23, 24]. Other studies suggested that IL-33 participated in the pathogenic process of acute hepatitis induced by Con-A [25, 26] and IL-33 overexpression was associated with the development of HBV/HCV-related liver fibrosis [27]. Another study found that serum IL-33 levels in patients with CHB were significantly higher than those in healthy controls. Besides, treatment with adefovir dipivoxil to inhibit the replication of HBV dramatically decreased the levels of serum IL-33 in patients with CHB. These confirmed that IL-33 could play a significant role in the progression of CHB and the data suggested that IL-33 might be a pathogenic factor in the pathogenic process of CHB patients, but no correlation was found between the levels of IL-33 and ALT or AST in their study. Further, serum ST2 levels were significantly higher in patients with CHB than those in healthy controls. Treatment with adefovir dipivoxil for 12 weeks did not significantly change the levels of serum ST2 [10]. A previous study had shown that serum ST2 levels in patients with acute liver failure were higher than those in patients with chronic liver failure and HC [1]. It was possible that high levels of serum ST2 were an early marker for liver injury, while high levels of serum IL-33 may be associated with the development and progression of liver fibrosis and damage [27]. Another study showed that the dendritic cells responded directly to IL-33 through ST2. The IL-33 and DC interaction may represent a new pathway to initiate Th2-type immune responses [28].

Our study showed that the serum levels of IL-33 and ST2 of the chronic hepatitis B virus carriers in immunotolerant phase were significantly lower than those of the HC (*P* = 0.007 for IL-33 and *P* = 0.039 for ST2). The serum levels of IL-33 and ST2 in patients with CHB ALT1ULN-2ULN group were lower when compared with those in the HC, however, the difference was not significant (*P* = 0.354 for IL-33 and *P* = 0.815 for ST2). The serum levels of IL-33 and ST2 in patients with CHB ALT ≥ 2ULN groups were significantly higher than those in the HC, respectively (*P* <0.05, respectively). Changes of serum IL-33 and ST2 levels were found to be positively associated with ALT levels in patients with chronic HBV infection (*rs* = 0.879, *P* < 0.001 for IL-33; *rs* = 0.923, *P* < 0.001 for ST2). However, there was no significant correlation between ALT and IL-33, or ST2 in healthy controls (*rs* = −0.134, *P* = 0.497 for IL-33; *rs* = −0.012, *P* = 0.952 for ST2). There were no significant differences in the serum levels of IL-33 and ST2 between patients with HBeAg-positive CHB and those with HBeAg-negative CHB. Our data provided evidence that serum levels of IL-33 and ST2 elevated with the increase of ALT levels in patients with CHB, and reduced in patients with CHB ALT1-2ULN and chronic hepatitis B virus carriers in immunotolerant phase.

There were several limitations with our study. We did not carry out any multivariate analysis of all parameters that might associate with serum IL-33 and ST2 levels due to the small sample size. Besides, we didn’t conduct liver biopsy so that we did not figure out the source for IL-33 and ST2 through histopathological examinations. Although more detailed studies were necessary to determine the role and mechanisms of IL-33 and ST2 in the pathogenic process of chronic hepatitis B virus infection, our novel findings might provide new insights into understanding the role of IL-33 and ST2 in the pathogenesis of chronic hepatitis B virus infection. We speculated that IL-33 and ST2 could be used as an indicator to judge the patient's condition, which could help doctors choose antiviral drugs for the patients and assess the therapeutic effects. We will conduct further study to investigate the changes of serum IL-33 and ST2 after antiviral treatment in future.

## Conclusions

In conclusion, our data indicated that serum levels of IL-33 and ST2 elevated with the increase of ALT levels in patients with CHB. The serum levels of IL-33 and ST2 varied in different courses of chronic hepatitis B virus infection. We surmised that IL-33 and ST2 might indicate liver damage of patients with chronic hepatitis B, just like ALT. They were associated with the natural course of chronic hepatitis B virus infection.

### Availability of data and materials

The raw data will be provided upon request by Dr. Kai Wang (Correspondence author), Email: wangdoc876@126.com; wangdoc2010@163.com.

## Abbreviations

ALT: alanine aminotransferase
CHB: chronic hepatitis B
ELISA: enzyme-linked immunosorbent assay
HBV: hepatitis B virus
HC: healthy control
IL-33: Interleukin-33
PCR: polymerase chain reaction
Th2: T helper-2
ULN: upper limit of normal
